# Keeping RelApse in Chk: molecular mechanisms of Chk1 inhibitor resistance in lymphoma

**DOI:** 10.1042/BCJ20220461

**Published:** 2022-11-23

**Authors:** Elizabeth M. Black, Yoon Ki Joo, Lilian Kabeche

**Affiliations:** 1 Department of Molecular Biophysics and Biochemistry, Yale University, New Haven, CT 06511, U.S.A.; 2 Yale Cancer Biology Institute, Yale University, New Haven, CT 06516, U.S.A.

**Keywords:** cell survival, DNA damage, DNA replication and recombination, inhibitor resistance

## Abstract

Chk1 is a member of the DNA damage response pathway, whose loss leads to replication stress and genome instability. Because of its protective role against lethal levels of DNA replication stress, Chk1 has been studied as a valuable and intriguing target for cancer therapy. However, one of the most prominent challenges with this strategy is development of resistance to Chk1 inhibitors, rendering the treatment ineffective. In their recent papers, Hunter and colleagues demonstrate multiple mechanisms by which Chk1 inhibitor resistance can arise in lymphomas. Specifically, this series of papers identify the relationship between dysfunction in NF-κB and the development of Chk1 inhibitor resistance through a loss of Chk1 activity in mouse models of lymphoma. They identify that cells lacking Chk1 activity can compensate for this loss through up-regulation of alternative pathways, such as PI3K/AKT. Finally, this work also identifies a novel role for Claspin, an important Chk1 activator, in female fertility and cancer development, furthering our understanding of how dysfunction in the Claspin/Chk1 signaling pathway affects disease states. These findings improve our understanding of drug resistance in cancer therapy, which has important implications for clinical use of Chk1 inhibitors.

Pharmacological inhibition of kinase activity has revolutionized the way we dissect the function of these proteins in the laboratory and treat cancers and other diseases in the clinic. Kinases control many essential cellular pathways, such as regulating cell division and protecting genome stability by ensuring that the genetic content in every cell is accurately replicated and segregated each division cycle. These pathways are often dysregulated in cancer, with many cancer cells showing aberrant proliferation and genome instability. Although these phenotypes have inspired several putative targetable vulnerabilities, high rates of genome instability and heterogeneity further challenge cancer treatment by allowing for selectable adaptive heterogeneity, such as by up-regulation of compensatory pathways [1,2]. Given these defects, cancer cells can become ‘addicted’ to pathways that maintain their viability, such as the DNA damage response (DDR) pathway, which detects and signals following DNA damage and replication stress. Pharmacologically targeting components of the DDR can then in turn exacerbate genome instability in cancer cells to a point where they can no longer survive.

This logic has inspired the use of Chk1 inhibitors in the clinic, as Chk1 is an essential effector kinase of the DDR pathway and protects cells against replication stress. Like other inhibitors, effective therapeutic application of Chk1 inhibitors is challenged by acquired drug resistance [3]. Thus, relapse from acquired drug resistance poses a continued and challenging problem for cancer therapy due to the inherent ‘whack-a-mole’ pattern of resistance, in which targeting one pathway leads to up-regulation of other pathways that promote cancer cell survival. Therefore, it is essential to fully investigate the mechanisms that drive inhibitor resistance and how other parallel pathways respond in cancer cells to improve the efficiency of cancer treatments targeting protein kinases.

To better understand how cancers with high levels of replication stress respond, and then eventually stop responding to, Chk1 inhibition, several papers in this issue by Hunter et al. and Madgwick et al. use mouse model systems to probe the molecular consequences of losing Chk1 activity and how these systems circumvent Chk1 activity to develop resistance.

The studies of Chk1 inhibitor resistance performed their experiments using a mouse model of lymphoma called Eµ-Myc, a common model of B-cell lymphoma in which the oncogene Myc is overexpressed [4]. The researchers chose this system because Eµ-Myc overexpression causes elevated levels of replication stress, recapitulating a context in which Chk1 activity is essential to avoid cell death. As expected, they observed that lymphomas that arise from this system are highly sensitive to treatment with Chk1 inhibitors [5–6]. However, when this system is further perturbed by genetic manipulation of NF-κB, an important transcriptional regulator that controls cellular proliferation and apoptosis, these tumors are no longer sensitive to Chk1 inhibition.

These papers tackle two key questions about resistance to Chk1 inhibitors: (1) how does it develop, and (2) how do cells adapt to loss of the target protein?

## How does Chk1 inhibitor resistance develop?

The researchers developed a model of Chk1 inhibitor resistance by combining the Myc overexpression background (Eµ-Myc) with mutations to different subunits of the NF-κB pathway: a genetic knockout of the cRel subunit (cRel^−/−^) [5] or a point mutation of threonine 505 in the RelA subunit (RelA T505A) [6]. Previous work identified that threonine 505 is a target of Chk1, and its phosphorylation negatively regulates NF-κB function [7,8]. In the presence of a wild-type NF-κB complex, Eµ-Myc lymphomas are highly sensitive to treatment with a Chk1 inhibitor. In contrast, when NF-κB is mutated with either the cRel^−/−^ or RelA 505A, these lymphomas are resistant to Chk1 inhibitor [5,6]. Interestingly, these papers determine that resistance to Chk1 inhibitor can be caused by at least two mechanisms: loss of the protein itself or loss of protein activity by down-regulation of an upstream activating protein.

**Figure 1.**
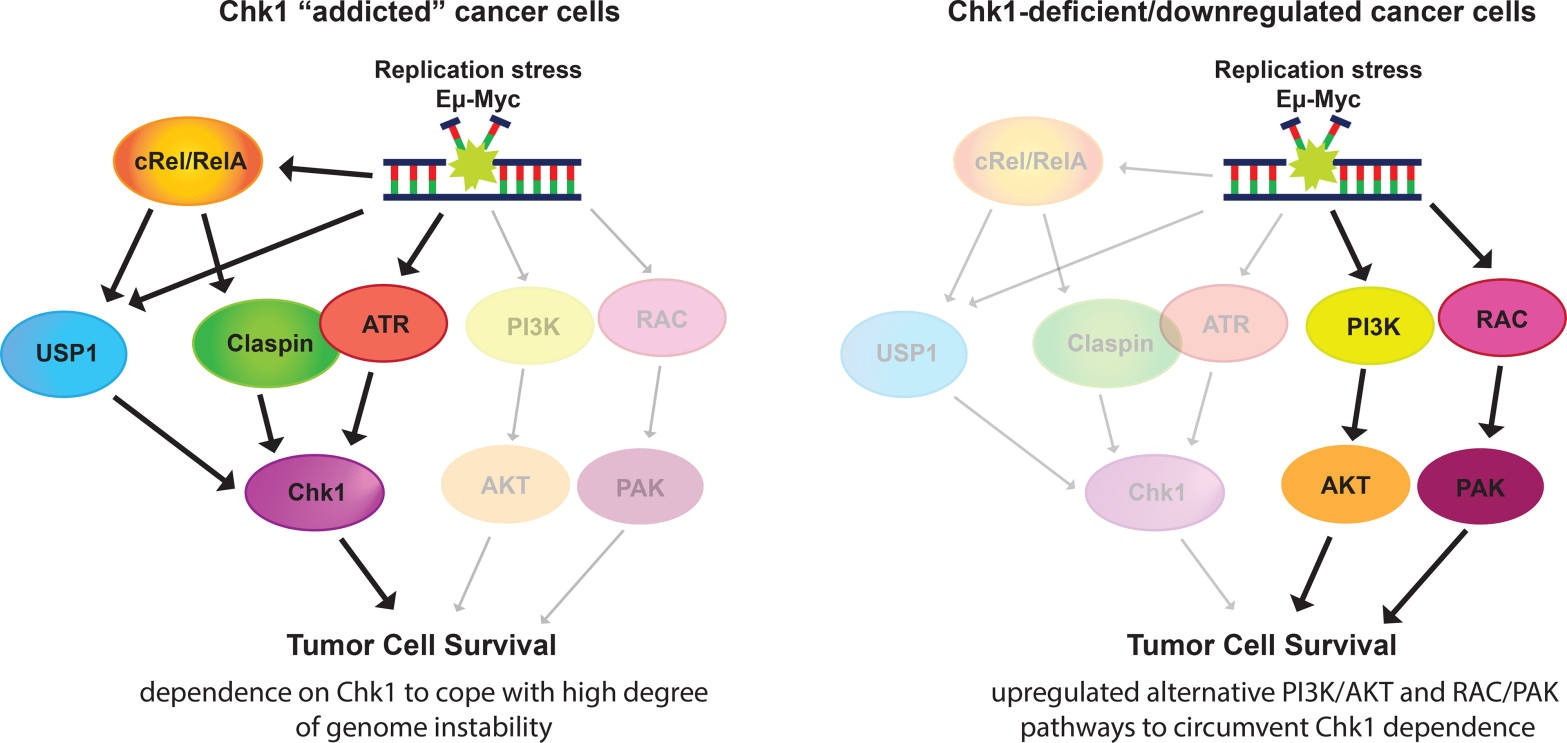
Model of Chk1 signaling in Eµ-Myc lymphomas with high levels of replication stress and subsequent dysregulation of Chk1 signaling in Eµ-Myc lymphomas with perturbations to the NF-κB subunits cRel or RelA. Following Chk1 degradation or inactivation when NF-κB is perturbed, the PI3K/AKT and RAC/PAK pathways are up-regulated to ensure tumor cell survival.

Hunter et al. profiled the phosphoproteome and transcriptome of Eµ-Myc lymphomas in an either wild-type or perturbed NF-κB complex genetic background that were treated with or without Chk1 inhibitor [5]. This experiment provides important insight into the mechanisms driving resistance in different genetic backgrounds. The authors determined that one major cause of Chk1 inhibitor insensitivity in the Eµ-Myc/*cRel*^−/−^ lymphomas is loss of Chk1 protein itself. Concurrent with a loss of Chk1 protein, these cells also significantly down-regulate USP1, a protein responsible for deubiquitinating Chk1 to prevent its degradation [5]. Taken together, these findings suggest that Chk1 inhibitor-resistant Eµ-Myc/*cRel*^−/−^ lymphomas develop resistance by down-regulating USP1, which results in Chk1 degradation, making it un-targetable (Figure 1). Importantly, the observation that *in vitro* evolved cell lines that develop Chk1 inhibitor resistance also lose USP1 expression suggests that this mechanism is a common and reproducible mechanism of Chk1 resistance [5].

In a separate study, the authors also identify a parallel mechanism to promote resistance to Chk1 inhibitors that is not loss of Chk1 itself, but instead causes reduced Chk1 activity. The researchers found that Eµ-Myc lymphomas containing a RelA 505A mutation have decreased expression at the mRNA level of Claspin [6], an important protein that promotes Chk1 activity following replication stress. The authors show that reduced Claspin expression leads to reduced Chk1 activity and decreases the efficacy of Chk1 inhibitors in this system [6].

This work provides important insight into how Chk1 abundance and activity is regulated to confer resistance to treatment with inhibitors. Future work may examine the mechanistic basis for USP1 and Claspin down-regulation in Eµ-Myc cells with genetic perturbations to NF-κB subunits. For example, are USP1 and Claspin direct transcriptional targets of NF-κB? Although Claspin has been described as a transcriptional target of cRel in a cell culture system [9], whether RelA directly regulates Claspin transcription in a mouse model of replication stress remains an open question. Additionally, could perturbations to NF-κB be indirectly affecting other cellular processes, such as DNA replication, that in turn put selective pressure on these cells to down-regulate Chk1 activity through USP1 or Claspin down-regulation? Determining how and why Chk1 is down-regulated when the NF-κB complex is perturbed will lend important insight into the molecular relationship between these proteins and help better inform the use of Chk1 inhibitors in cancers with NF-κB mutations.

## How do cells adapt to loss of Chk1?

In the third paper in this series, Hunter et al. further examine the data from the large-scale phosphoproteomic experiment to determine how Chk1-deficient cells survive despite the high levels of replication stress present in the Eµ-Myc background. Chk1 is an essential protein that protects against elevated levels of replication stress. How, then, are cells that lack Chk1, such as Eµ-Myc cRel^−/−^, surviving without Chk1?

Close examination of the phosphoproteomic and transcriptomic data reveals that the up-regulated (phospho)peptides in Chk1-inhibited wild-type samples have many peptides that are similarly up-regulated in cRel^−/−^ lymphomas, which lack Chk1 protein [5] (see above). This experiment therefore provides a rich dataset to interrogate the cellular consequences of Chk1 loss by either pharmacological inhibition or loss of the protein.

Interestingly, Eµ-Myc cRel^−/−^ lymphomas have significant up-regulation of a pathway involving PI3K and AKT kinases, which are known to inhibit apoptosis and promote cell survival, providing an elegant model to explain how these cells, which lack Chk1 protein, can survive elevated levels of replication stress [10]. In a complementary model of NF-κB dysfunction, Eµ-Myc RelA 505A mutant cells, which also lack Chk1 activity, up-regulate the RHO/RAC/PAK pathway. Given the well-studied role for this pathway in promoting cellular proliferation, inhibiting apoptosis, and metastasis [11], the researchers hypothesize that up-regulation of the RHO/RAC/PAK pathway is sufficient to promote cell survival in the absence of Chk1 signaling following DNA replication stress [10]. Importantly, in both models of Chk1 inhibitor resistance, inhibition of their respective up-regulated pathways using small molecule inhibitors significantly decreases lymphoma size, suggesting a clinical target for Chk1 inhibitor-resistant cancers. One interesting implication of this work is that inhibitors against Chk1 and PI3K may be used synergistically to prevent Chk1 inhibitor resistance from developing, although future work will be necessary to test this hypothesis.

On a molecular level, the sequence of Chk1 inhibitor resistance development and up-regulation of compensatory pathways is currently unknown. Is up-regulation of these pathways a prerequisite for Chk1 down-regulation and the development of Chk1 inhibitor resistance, or is this a secondary effect of Chk1 loss? Does up-regulation of these pathways minimize the replication stress in Eµ-Myc lymphomas? This information would be important for the development of biomarkers for Chk1 inhibitor sensitivity and resistance to improve the clinical application of Chk1 inhibitors in cancer therapy.

## New perspectives on relationship between Claspin and cancer initiation and development

Claspin, like Chk1, is an important protein in the DDR pathway that controls the cellular response to DNA replication stress [12]. Although Claspin is a well-appreciated activator of Chk1 [13], its relationship to disease progression in humans is unclear. The researchers reasoned that previous reports correlating increased Claspin expression in cancers may be adaptive to promote cell survival in response to the high degree of replication stress, rather than suggesting a direct role of Claspin in disease severity. To tease apart these effects, Madgwick et al. characterized a Claspin heterozygous knockout mouse. They observed that mice lacking Claspin have a myriad of defects, such as impaired female fertility and an increase in the disease states lymphoid hyperplasia, non-alcoholic fatty liver disease, and hepatocellular carcinoma following liver damage by partial hepatectomy or the DNA-damaging agent DEN [14]. This work establishes Claspin as a tumor suppressor, particularly in the model of cancer initiation during recovery from damage to liver tissue. This experimental model of Claspin haploidy establishes Claspin as a pleiotropic regulator of normal physiology and cancer initiation and development. When taken together with work from Hunter et al. demonstrating Claspin loss in Eµ-Myc RelA 505A lymphomas, this suggests that these cells are not only more likely to become malignant, but would also be resistant to Chk1 inhibitor treatment, further challenging treatment of these cancers.

In summary, the work from Hunter et al. demonstrates the causes and consequences of Chk1 inhibitor resistance in Eµ-Myc-driven lymphomas with mutations in NF-κB. The broad principles from these studies, however, may be generalizable to other models of inhibitor resistance. This body of work not only furthers our understanding of the molecular relationship between Chk1 and NF-κB and how defects in the Claspin–Chk1 signaling pathway promote disease etiology, but also how drug resistance influences cancer evolution and offers clinicians important biomarkers for disease resistance. This work is a large step forward in the practical application of Chk1 inhibitors in the clinic and serves as a broad case study for other models of resistance.

## Open Access

Open access for this article was enabled by the participation of Yale University in an all-inclusive *Read & Publish* agreement with Portland Press and the Biochemical Society.
